# Effects of Dietary Vitamin E on Fertility Functions in Poultry Species

**DOI:** 10.3390/ijms16059910

**Published:** 2015-04-30

**Authors:** Deivendran Rengaraj, Yeong Ho Hong

**Affiliations:** Department of Animal Science and Technology, Chung-Ang University, Anseong, Gyeonggi-do 456-756, Korea; E-Mail: deivendran@cau.ac.kr

**Keywords:** vitamin E, fertility functions, poultry species

## Abstract

Vitamin E is found in high quantities in vegetable oils. Although vitamin E has multiple functions in humans and animals, its key function is protecting cells from oxidative damage. Since its discovery, several studies have demonstrated that vitamin E deficiency causes impaired fertility in humans and lab animals. However, the effects of vitamin E deficiency or of its supplementation on the fertility of farm animals, particularly on poultry, are less well studied. Therefore, a comprehensive review of the effects of dietary vitamin E on the fertility of poultry species is needed in order to understand the beneficial role of vitamin E in the maintenance of sperm and egg qualities. Based on the observations reviewed here, we found that a moderate amount of vitamin E in poultry diet significantly protects semen/sperm qualities in male birds and egg qualities in female birds via decreasing the lipid peroxidation in semen/sperms and eggs. This review provides an overall understanding of the effects of dietary vitamin E on fertility functions in poultry species.

## 1. Introduction

Vitamins are vital organic compounds that can be obtained in high quantities from vegetable oils. Vitamin tablets are also commercially available at a reasonable price. To date, thirteen vitamins have been discovered, named alphabetically vitamin A to vitamin K. The fat-soluble vitamin E was discovered in the early 1920s by Evans and Bishop [1]. Vitamin E is a generic term for a group of tocopherols and tocotrienols that have some amount of vitamin activity. Among the four tocopherols (α, β, γ and δ) and four tocotrienols (α, β, γ and δ) discovered, α-tocopherol is the most biologically active form and available in high quantities from vegetable oils, unprocessed cereal grains, and nuts [2,3,4,5]. Since the first discovery of vitamin E, numerous investigators have demonstrated the nutritional value of vitamin E in humans as well as in laboratory and farm animals. Vitamin E is absorbed via the lymphatic pathway and transported into the systemic circulation in association with chylomicrons [5]. After absorption, vitamin E is stored chiefly in the liver. Because of its fat-soluble properties it is incorporated in lipid storage organelles and plasma membranes, therefore it is also widely distributed throughout the body [2,5,6]. Vitamin E interacts with several other dietary components, including selenium, polyunsaturated fatty acids, sulphur-containing amino acids, other vitamins and minerals, and synthetic antioxidants [7,8,9,10,11]. Vitamin E has multiple critical functions in animals. For instance, it acts as an efficient biological antioxidant in protecting cells from the adverse effects of reactive oxygen species or free radical initiators [7,8,10,12]. It is required for the regulation of heme biosynthesis, apparently by controlling induction and repression of aminolevulinic acid synthase and porphobilinogen synthase [12]. Vitamin E plays a specific role in the essential transport of amino acids and possibly lipids in the intestine [7]. Vitamin E is also involved in iron metabolism and steroidogenesis [12], and it stimulates humoral and cellular immune responses against infectious diseases [13].

The symptoms and disorders of vitamin E deficiency vary, depending on the species affected. They include disorders of the nervous system, skeletal system, circulatory system, muscular system, cardiovascular system, immune system, and reproductive system [2,10,13]. Furthermore, vitamin E deficiency may manifest itself in a number of disorders of liver, kidney, and lung, and in adipose tissue [2]. Vitamin E deficiency may increase the risk of ischemic heart disease, breast cancer, and the incidence of infections [14], and it promotes susceptibility to dietary and environmental stress in humans and animals [7,13]. Vitamin E is clinically administered to treat several conditions and diseases in humans, and complications associated with high dose administrations have been reported [4,14]. Compared to observations in humans, data from animal studies show that vitamin E toxicity is low and that the vitamin is not mutagenic, carcinogenic, or teratogenic [4].

The activity of vitamin E was first identified as an essential dietary factor for male and female reproduction in rats [1,15]. Although vitamin E has a broad range of functions in the body, it is primarily crucial for fertility in humans and livestock species, including poultry. Generally, it has been shown that vitamin E deficiency causes abnormal spermatogenesis in males and failure to retain zygotes and fetal resorption in females [5,10,12]. Reproductive functions are crucial for healthy offspring and species survival of all animals, including poultry. Thus, the objective of this paper is to review studies that have examined the effects of dietary vitamin E on male and female fertility in poultry species.

## 2. Vitamin E in Poultry Nutrition

**Figure 1:**
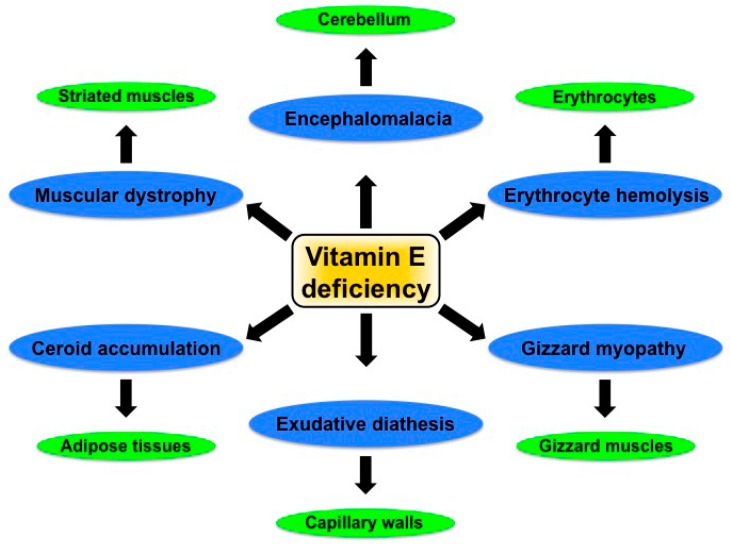
Commonly known vitamin E deficiency diseases and most vulnerable tissues in poultry species.

Because vitamin contents vary in western diets, it is important to know the recommended allowances of vitamins for humans [16]. However, due to many variables, it is very difficult to estimate the optimum allowances of vitamins for laboratory animal and livestock diets [15]. Dietary supplementation with vitamin E increases the resistance of animals against infectious diseases and is thus recommended for farm animals including poultry, swine, sheep, and cattle, to meet the increasing demand for meat, eggs, and milk [13]. The rearing of domesticated birds including chickens, quail, turkeys, ducks, and geese in poultry farms has been increasing worldwide for the purpose of meat and egg production. Among the poultry species, chickens are reared in huge numbers, *i.e.*, several billions. Nowadays, modern poultry feeds are largely supplemented with proteins, carbohydrates, lipids, minerals, and vitamins to meet the basal nutritional requirements for raising and maintaining healthy birds. The standard dietary supplementation with proteins, carbohydrates, lipids, minerals, and vitamins supports normal growth of birds. Similarly, the standard supplementation with certain minerals and vitamins helps to increase the birds’ immune resistance against pathogenic diseases [17,18,19,20]. The dietary requirement for vitamin E in poultry feed is highly variable and depends on the concentration and type of fat in the diet, the concentration of selenium, and the presence of pro-oxidants and antioxidants [21]. The National Research Council’s Committee on Animal Nutrition, USA, provided the nutrient requirements for poultry species including chickens, turkeys, geese, ducks, pheasants, and quail. According to its recommendations, poultry feed can be supplemented with 10 IU of vitamin E per kg feed (1 IU = 0.67 mg *dl*-α-tocopheryl acetate) for chickens aged up to six weeks, 5 IU/kg feed for chickens aged over six weeks, 12 IU/kg feed for turkeys aged up to eight weeks, and 10 IU/kg feed for turkeys aged over eight weeks. For ducks and Japanese quail, feed can be supplemented with 10 IU/kg feed and 12 IU/kg feed, respectively, for starting and growing birds. The dietary recommendations of vitamin E for poultry species during laying and breeding vary slightly [21]. Vitamin E is one of the essential nutrients in poultry feed, and its deficiency causes a wide variety of disorders in poultry species (Figure 1). These include nutritional muscular dystrophy that affects striated muscles, erythrocyte hemolysis that affects erythrocytes, and exudative diathesis that affects capillary walls. Furthermore, vitamin E deficiency can lead to membrane lipid peroxidation, affecting hepatic mitochondria and microsomes, as well as to an accumulation of ceroid in adipose tissues, and to cerebellar encephalomalacia in chickens [7,10,12]. In addition, vitamin E deficiency impairs feather development in chickens [10]. Vitamin E deficiency causes gizzard myopathy in turkeys and ducks, and an accumulation of ceroid in turkeys [10].

## 3. Effects of Dietary Vitamin E on Male Fertility Functions in Poultry Species

Since the first study on reproductive functions in rats by Evans and Bishop [1], several investigators have demonstrated the benefits of dietary supplementation with vitamin E on fertility in different animal species. In this chapter, we will review the effects of vitamin E supplementation specifically on fertility functions in male birds, based on evidence provided by several significant studies. Male fertility is principally related to semen and sperm qualities, including the volume of semen, concentration of sperms in the semen, sperm viability, sperm motility, sperm forward progression, and the sperm fertilizing capacity. These qualities can be affected by several environmental factors and endocrine disrupting chemicals, which may enter the body through diet, respiration, and skin contact [22]. The resulting adverse effects may be partially controlled or averted by clinical treatments or by dietary supplementation with certain beneficiary compounds, including minerals, vitamins, and antioxidants. Linoleic acid is one of the essential polyunsaturated fatty acids that cannot be produced *de novo* in vertebrates. If an animal diet is rich in any polyunsaturated fatty acids, the addition of an antioxidant is required in order to avoid peroxidation of the fatty acids [23]. Arscott and colleagues [24] fed adult male White Leghorn (WL) chickens with diets containing high levels of linoleic acid (7.3%) and an either low (4.3 mg/kg feed) or high (166.3 mg/kg feed) amount of vitamin E for 25 weeks. The males fed with diets high in linoleic acid and low in vitamin E showed impaired fertilizing capacity and sperm concentration in the semen. However, diet supplementation with a high amount of vitamin E prevented these adverse effects. The semen volume, hatchability of fertile eggs, and weight of the testes were not affected by any of the above diets. Furthermore, the authors suggested that vitamin E acts as an antioxidant that protects chickens against encephalomalacia by preventing the breakdown of linoleic acid to 12-oxo-*cis*-9-octadecenoic acid (keto acid). Encephalomalacia is a vitamin E deficiency disease, which readily occurs in chickens fed on a diet containing high levels of polyunsaturated fatty acids of the linoleic acid series and low levels of vitamin E [25]. In another study, Arscott and Parker [26] fed male chickens with a diet high in linoleic acid (7.3%) and low in vitamin E (4.3 mg/kg diet) from hatch to 28 weeks of age, and high in both linoleic acid (7.3%) and vitamin E (166.3 mg/kg diet) from 28 to 40 weeks. As a result, the adverse effects on fertility and semen concentration were restored in chickens fed on the diet high in vitamin E. Thus, the adverse effects of linoleic acid on male fertility are not permanent but can be reversed by vitamin E supplementation. Another compound, dilauryl succinate, does not contain peroxide or polyunsaturated fatty acids, however, similar to linoleic acid, it induces encephalomalacia in chickens. In a study by Yoshida and Hoshii [27], fertility of WL roosters fed on a diet containing 12% dilauryl succinate for 16 weeks was significantly low. When the roosters were fed a diet containing 12% dilauryl succinate and 200 mg/kg *dl*-α-tocopheryl acetate, the sperm fertilizing capacity was significantly high. Avian spermatozoa are rich in polyunsaturated fatty acids, in particular in docosatetraenoic acid and arachidonic acid [28,29]. The high proportions of polyunsaturated fatty acids provide membrane flexibility, which is essential for sperm mobility and sperm-egg fusion [30]. However, because of their high levels of polyunsaturated fatty acids, avian sperms are also very sensitive to reactive oxygen species, causing male infertility [28,31,32]. Therefore, an increased antioxidant status in semen or spermatozoa is a prerequisite for the prevention of male infertility. In a study by Surai and colleagues [31], six-month-old male Rhode Island Red chickens were fed with a diet containing 0, 20, 200, or 1000 mg/kg α-tocopheryl acetate for eight weeks. During the final two weeks, they observed that the concentration of vitamin E in semen and sperms had doubled, and the susceptibility of the semen to lipid peroxidation was decreased, in particular in birds fed with 200 mg/kg of vitamin E. In another study [33], 30-week-old WL roosters were fed with a basal diet high in fish/soybean oil and with or without supplementation of vitamin E (30, 200, or 400 mg/kg). After 38 weeks, the roosters fed with fish/soybean oil showed the lowest total antioxidant status in the semen. However, the addition of vitamin E to the fish/soybean oil diet resulted in an increase in semen volume, motility, and sperm potency at 38 weeks. Zaniboni and colleagues [32] allowed male turkeys to feed on a basal diet supplemented with 60 mg/kg of α-tocopheryl acetate from 26 to 39 weeks of age. From 40 to 60 weeks, the turkeys were allowed to feed on the basal diet additionally supplemented with 60 mg α-tocopheryl acetate and 2% fish oil. As a result, the α-tocopheryl status in the turkey semen was increased two-fold. Biswas and colleagues fed Indian native Kadaknath male chicks with a basal diet supplemented with 10, 100, or 200 mg/kg *dl*-α-tocopheryl acetate from hatch to 30 weeks of age. An analysis performed during the final three weeks of the trial revealed that the proportion of abnormal and dead spermatozoa was significantly lower and the fertilizing capacity was significantly higher in birds fed with 100 mg/kg of vitamin E. Moreover, the vitamin E status of semen and spermatozoa was higher in birds fed with 100 mg/kg of vitamin E than that in birds fed with 10 mg/kg of vitamin E [34]. These studies suggest that an increased antioxidant status of semen or sperm is based on an increase in the antioxidant content of the diet. In another study, Biswas and colleagues fed male Japanese quail with a basal diet containing 15 IU, 150 IU, or 300 IU of *dl*-α-tocopheryl acetate per kg feed, from the age of one day for up to 25 weeks. During these dietary trials, plasma testosterone concentration, testicular weight, as well as semen volume were not significantly changed. However, the percentage of abnormal and dead sperms was significantly lower, and the sperm capacity was higher in quail fed with a diet containing 150 IU/kg of vitamin E [35]. In a similar study, Hooda and colleagues fed male Japanese quail with a basal diet supplemented with different concentrations of vitamin E (0–300 IU *dl*-α-tocopheryl acetate/kg feed) from the age of five weeks onwards, for 10 to 13 weeks. Among the different concentrations of vitamin E, 75 IU/kg feed elicited the best result in protecting the male fertility functions in quail [36]. Taken together, these studies, in particular those by Surai *et al.*, Biswas *et al.*, and Hooda *et al.* suggest that a moderate level of vitamin E is best for maintaining the fertility functions in male chickens and quail [31,34,35,36].

Selenium is a trace element and frequently added to animal diet as a supplement for the maintenance of reproductive functions, and a deficiency in dietary selenium causes a decrease in sperm concentration, sperm motility, and sperm capacity in humans, lab animals, and farm animals, including poultry species [37,38]. Selenium is a component of selenoproteins, such as glutathione peroxidase, which protect sperms against oxidative damage [39,40]. It is well known that vitamin E interacts with selenium, and that both play a role in the maintenance of reproductive functions as well as in the reduction of reactive oxygen species and free radical generation [7,8]. Surai and colleagues fed Rhode Island Red males with a balanced diet supplemented with selenium (0.3 mg/kg diet) and vitamin E (20 or 200 mg/kg diet) and observed that the activity of glutathione peroxidase in testes, semen, and sperms was significantly increased [41]. Compared to chickens and quail, geese exhibit lower semen quality, egg production, and fertility and hatchability rates. When three-year-old White Koluda male geese were fed with a control diet and an experimental diet supplemented with 0.3 mg/kg feed of selenium and 100 mg/kg feed of vitamin E, the semen volume and sperm concentrations in the semen were significantly increased (1.5- and 1.7-fold, respectively) during the next reproductive season, in the males fed on the experimental diet. Also, the level of lipid peroxidation was significantly lower in males fed on the experimental diet [42]. The study by Jerysz and Lukaszewicz suggests that combined supplementation with selenium and vitamin E enhances the reproductive functions of certain avian species that naturally show low reproductive behavior. Antioxidant supplementation is not only important for lipid-containing poultry feed but also a prerequisite for any basal diet in order to maintain fertility. In a study by Lin and colleagues [43], freshly hatched Taiwan Native male chicks were fed with maize/soybean diets for up to 23 weeks of age. After 23 weeks, the birds were allowed to feed on maize/soybean diets supplemented with 80 mg/kg feed of *dl*-α-tocopheryl acetate for up to 52 weeks of age. Males fed with the vitamin E supplemented diet showed significantly higher sperm viability, sperm motility, and sperm concentration in the semen. This study suggests that a maize/soybean containing diet is inadequate for maintaining the fertility functions in male birds, thus vitamin E supplementation is required.

Molting in birds is a periodic occurrence, during which, at least once a year, the feathers are replaced. Molting is commonly associated with hens. However, several studies reported that molting in males might lead to an improved semen quality [30,44]. Molting in male birds can be experimentally induced by withdrawal of feed, water, or both, or by dietary manipulation with imbalanced minerals such as sodium, calcium, iodine, and zinc [30,44]. Khan and colleagues [45] induced molting in WL male birds with a basal diet containing 3000 mg zinc oxide per kg feed. After two weeks of molting, the birds were fed with either a basal diet or a diet supplemented with vitamin E (100 IU/kg feed). At least three weeks after the trial, the semen volume, sperm motility, and sperm capacity in fertilizing eggs had significantly increased in birds fed with the vitamin E supplemented diet, compared to the control group. Several investigators have demonstrated the association of vitamin E deficiency with impaired male reproductive functions. It has been identified as an anti-sterility vitamin, and its deficiency or long-term omission in the diet causes abnormal spermatogenesis and affects the semen qualities, including sperm viability, sperm motility, and sperm capacity [5,10,12]. Earlier studies reported that testicular degeneration resulted when Rhode Island Red males were fed a vitamin E deficient diet for two years [23,24]. From the studies reviewed above, it has been understood that dietary supplementation with vitamin E protects semen quality, especially by preventing the breakdown of polyunsaturated fatty acids, which can cause oxidative damage. The recommended amount of vitamin E in poultry diet for maintaining male fertility functions can vary, depending on the investigator. For instance, Khan *et al.* recommended that a poultry diet should contain at least 10 mg vitamin E per kg of feed [45]. However, several studies recommended that a poultry diet should contain a moderate amount (not very low and not very high) of vitamin E [31,34,35,36].

## 4. Effects of Dietary Vitamin E on Female Fertility Functions in Poultry Species

This chapter will review the effects of dietary vitamin E supplementation on female fertility functions in different poultry species, based on the evidence provided by several significant studies. The fertility functions of females, like those of males, are crucial for successful production of healthy offspring. More specifically, in poultry species the daily egg production, the egg quality, including egg weight and components of the yolk and albumin, and the egg fertility and hatchability are the most important factors that determine healthy offspring. The number of fertile eggs produced for hatching dictates the ultimate profitability of hens [46]. The nutrients required for embryo development are derived mainly from the yolk and albumin stored in the eggs. A chicken egg contains significant amounts of nutrients, including carbohydrates, proteins, lipids, vitamins, and trace elements, and these nutrients can be increased or decreased in eggs by altering the dietary composition. The concentrations of total lipids, including the polyunsaturated fatty acids of the linoleic acid series, and of antioxidants are relatively stable in the eggs of chickens fed with a standard diet, but they are subject to alteration by major changes in dietary nutrient composition [47,48]. Similar to Arscott’s study in males [24], Machlin and colleagues [23] fed WL hens with a basal diet high in linoleic acid (7%) and low in vitamin E (20 IU/pound feed) for eight weeks. By the end of this period, egg production had decreased from 78% to 25%. Only 37% of the eggs were fertile, and none of the fertile eggs was hatched. When hens were fed with a diet containing high amounts of both linoleic acid (7%) and vitamin E (100 IU/pound feed) for eight weeks, the egg production reached an average of 57%. Seventy-six percent of the eggs were fertile and 67% of the fertile eggs were hatched. Together with the results obtained for males [24], this study suggests that vitamin E acts as an antioxidant, in that it protects female fertility by preventing the breakdown of linoleic acid, which would then lead to oxidative damage. Additionally, the study by Machlin and colleagues showed that hens fed with diets low in linoleic acid do not require additional vitamin E or antioxidants for maintenance of egg production, fertility, and hatch of fertile eggs [23]. When Yoshida and Hoshii fed WL hens with a basal diet containing 12% dilauryl succinate, which has similar effects as linoleic acid, the fertility of eggs and the hatchability of fertile eggs were observed to be low. However, these effects were prevented by adding 200 mg/kg feed of *dl*-α-tocopheryl acetate to the same diet, containing 12% dilauryl succinate [27].

Vicine, an alkaloid found in high quantities in faba beans (*Vicia faba* L.), has a marked influence on the metabolism of laying hens. Dietary vicine causes peroxidation of cellular components, resulting in abnormal lipid transport and a decrease in fertility. In a study by Muduuli *et al.*, laying WL hens fed with a vicine containing diet showed decreased egg weight, fertility, and hatchability. However, vitamin E supplementation slightly increased the egg weight and markedly protected fertility and hatchability of eggs [49]. This study clearly indicates that the antioxidant property of vitamin E is very helpful in reducing the adverse effects of toxic compounds like vicine. Similarly to the observations reviewed above, an adequate amount of vitamin E was found necessary in the poultry diet for maintaining female fertility, regardless of whether or not the diet is high in polyunsaturated fatty acids. In a study by Lin *et al.* [48], hatched Taiwan Native female chicks were fed with a maize/soybean diet for up to 17 weeks of age. After 17 weeks, the birds were allowed to feed on a maize/soybean diet supplemented with 80 mg/kg feed of *dl*-α-tocopheryl acetate for up to 46 weeks of age. As a result, hens fed with the vitamin E supplemented diet showed a better performance in egg production and egg mass. Likewise, vitamin E supplemented diet showed a better performance in fertility (over 7%) and hatchability (over 13%). In another study, Hooda and colleagues fed five-week-old male and female Japanese quail with a basal diet containing different concentrations of vitamin E (0, 75, 150, 225, and 300 IU *dl*-α-tocopheryl acetate/kg feed), during a period of 5 to 13 weeks of age. Then, the males and females were allowed to mate in different groups: control males with control females, control males with females on vitamin E supplemented diets, and control females with males on vitamin E supplemented diets. As a result, males paired with females after both had been fed with a vitamin E supplemented diet led to slightly better fertility and hatchability of quail eggs. However, these results were not influenced by an increase in dietary vitamin E [36]. When Japanese quail were maintained on a soybean meal diet containing low levels of vitamin E (2 IU/kg diet) for 35 weeks, the percentage of fertile eggs was significantly decreased and the hatchability of fertile eggs was severely affected. However, no other clinical symptoms were observed [50]. Kling and Soares suggested that this much lower amount of vitamin E in the diet is extremely inadequate for supporting normal reproduction in quail.

**Figure 2:**
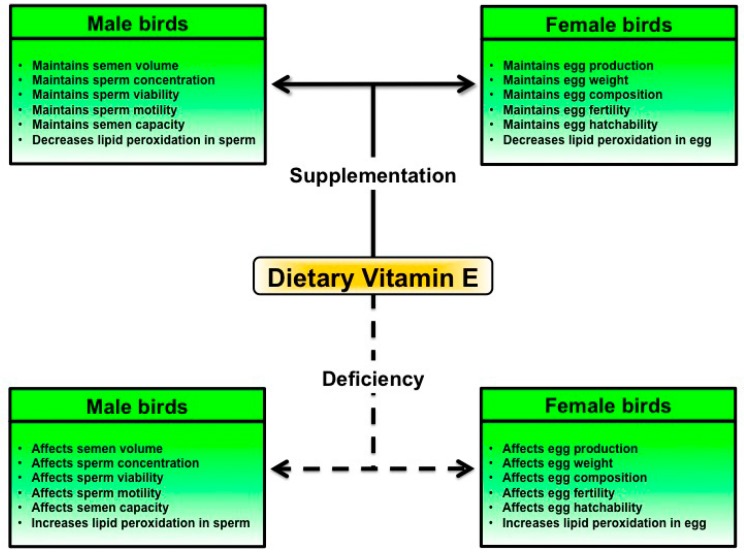
The main effects of dietary vitamin E supplementation on male and female fertility functions in poultry species.

In avian species, the sperms are stored for a short period in male reproductive tract (vas deferens). Following natural mating or artificial insemination, the ejaculated sperms undergo a selection process and the selected sperms are stored for a prolonged period in female reproductive tract at two sperm storage sites [51,52,53]. The primary storage site is found in the utero-vaginal junction, and the secondary storage site is found in the lower portion of the infundibulum [52,53]. Studies by Breque and colleagues indicate a positive effect of dietary vitamin E on the antioxidant status of sperm storage sites in hens [53]. In avian species, the proportion of sperms found particularly at utero-vaginal junction significantly correlate with the sperms found at perivitelline layer of eggs [52,54,55]. Moreover, Brillard and Antoine suggest that hen’s egg achieves 100% of fertility when the perivitelline layer contains 1440 or more sperms [54]. Taken together, it is well understood that vitamin E deficiency significantly affects the fertility of female poultry species [5,12]. If the hatched chicks show vitamin E deficiency symptoms, the amount of vitamin E in eggs should be increased through supplementation of the maternal diet. In addition, the amount of vitamin E in the diet should be correlated with the amount of dietary selenium and polyunsaturated fatty acids. If the poultry diet contains a sufficient amount of selenium and is not rich in polyunsaturated fatty acids, about 20 mg vitamin E per kg diet is required for maintaining fertilization rate of breeding hens [56]. In addition, Yoshida and Hoshii recommended an amount of more than 500 μg vitamin E per egg to ensure the hatching of healthy chicks [27]. The main effects of dietary vitamin E supplementation on male and female fertility in poultry are shown in Figure 2. From analyses of male and female birds, it has been understood that feeding the birds with at least 100 mg vitamin E per kg diet can prevent vitamin E deficiency problems.

## 5. Conclusions and Future Perspectives

In this communication, we reviewed the effects of dietary antioxidant vitamin E on fertility of male and female poultry species. The review suggests that a moderate vitamin E supplementation of a balanced poultry diet significantly maintains male fertility functions, including semen volume, sperm concentration, sperm viability, sperm motility, and sperm capacity, in poultry species. In addition, a moderate vitamin E diet supplementation significantly maintains female fertility in poultry species, including egg production, egg fertility, and egg hatchability. It has been understood that the maintenance of fertility in male and female birds by vitamin E is based on its property as a defense mechanism against oxidative damage, which predominantly results from the breakdown of polyunsaturated fatty acids. Furthermore, the deficiency or long-term omission of vitamin E in the diet impairs male and female fertility in birds. Based on the information discussed here, this review suggests a few future perspectives for research on poultry species. (1) Most studies referring to poultry species have focused on the effects of dietary vitamin E on semen and sperm qualities in males, and egg qualities in females. However, the effects on Leydig cells and Sertoli cells in males, and on theca cells and granulosa cells in females need to be elucidated; (2) The effects of vitamin E deficiency or supplementation on male and female steroidogenesis are not well understood; (3) The effects of vitamin E deficiency or supplementation on the functions of fertility-associated genes and proteins need to be elucidated; (4) Most studies recommended a moderate amount of vitamin E (75–100 mg/kg diet) for the maintenance of fertility functions in male and female birds. In agreement with these suggestions, the National Research Council’s Committee on Animal Nutrition should establish a recommended allowance; (5) Most studies referring to the fertility of poultry species have focused on the interaction of vitamin E with polyunsaturated fatty acids and selenium, however, the interaction of vitamin E with other vitamins and minerals are less well known. Collectively, this review provides an overall understanding of the effects of dietary vitamin E on fertility of diverse poultry species.
